# Awareness and perception of postpartum depression among rural Indian mothers

**DOI:** 10.1007/s44192-026-00553-x

**Published:** 2026-07-23

**Authors:** Seema Hemendra Satbhai, Shamim Mohammad

**Affiliations:** School of Health Sciences and Technology, Dr. Vishwanath Karad MIT World Peace University, Pune, Maharashtra 411038 India

**Keywords:** Postpartum depression, Awareness, Rural mothers, India

## Abstract

Postpartum depression (PPD) is a major yet neglected public health concern that affects maternal and child health outcomes. Awareness of PPD is crucial for early detection and timely help-seeking; however, community-level awareness remains limited in India. This cross-sectional study assessed awareness of PPD and attitudes towards help-seeking behaviour related to PPD among postpartum mothers in rural Pune using a structured questionnaire. A total of 220 postpartum mothers between 6 weeks and 6 months post-delivery were recruited through consecutive sampling during immunisation sessions at Primary Health Centres (PHCs) in Haveli Taluka, Pune. Of the 220 participants, 57 (25.9%) had heard of PPD. Social media platforms (61.4%) were the primary source of information. Nearly half (50.9%) of the mothers who had heard of PPD perceived it as a “normal part of motherhood.” Inferential analysis indicated that awareness of PPD was significantly associated with maternal age and education level. The findings highlight low awareness of PPD and underscore the need to integrate maternal mental health literacy into routine community health education and antenatal counselling to improve early recognition and care-seeking.

## Introduction

Postpartum depression (PPD) is a major but often under-recognised and neglected public health concern affecting nearly 18% of mothers globally [1]. PPD is a form of perinatal depression that begins during pregnancy or within the first year after childbirth [2], and if left undetected, can adversely affect maternal functioning, infant development, bonding and long- term family well-being [3].

In India, the reported prevalence varies across studies, estimating a pooled prevalence of 22%with higher rates in urban areas (24%) and elevated rates in rural areas (17%) [4]. However, evidence remains limited, and the burden may be underestimated due to poor detection, stigma, and lack of awareness. Previous qualitative research from India has shown that cultural expectations around motherhood often prevent women from acknowledging emotional distress, as postpartum suffering is perceived as a normal social experience rather than a mental health condition requiring care [5]. These norms contribute to the stigma and low recognition of PPD among mothers.

Awareness and mental health literacy are critical first steps in early detection and in promoting appropriate help-seeking behaviour [6, 7].

PPD remains largely unrecognised in rural India due to limited mental health literacy, inadequate awareness of symptoms, stigma and poor integration of maternal health services within routine maternal care [8–12].

Many women across Indian and international settings perceive emotional distress, crying, irritability, or withdrawal as a “normal” part of motherhood, shaped by cultural expectations, household responsibilities, and traditional gender roles [8, 13–15].

When mothers are unaware that PPD is a medical condition, they may be less able to identify their symptoms or seek appropriate care and support [8, 15, 16].

Limited awareness and poor symptom recognition may contribute to delayed help-seeking and under-recognition of postpartum psychological distress [8, 15, 16].

Despite the growing evidence on PPD prevalence in India, awareness-related evidence is extremely limited [11, 17], particularly in rural communities where changing family structures and increasing maternal workloads may influence the availability of postpartum support systems [18, 19]. To the best of our knowledge, very few studies from India and none from rural Pune have systematically assessed mothers’ awareness, understanding of symptoms, and help-seeking perceptions regarding PPD [17]. Therefore, assessing PPD awareness is essential for identifying gaps that prevent early recognition and timely care. Accordingly, the present study was undertaken to assess the awareness of PPD among postpartum rural mothers as the primary outcome and to explore symptom recognition, sources of information and attitudes towards help-seeking. These findings can inform maternal mental health literacy initiatives and help integrate PPD awareness into routine antenatal and postnatal care.

## Methodology

### Study design and settings

This community-based descriptive cross-sectional study was conducted from January 2025 to May 2025 in rural areas of Haveli Taluka, Pune District, Maharashtra, as part of a large research project. Data were collected from postpartum mothers attending weekly immunisation sessions conducted at subcentres under Khadkwasla Primary Health Centre (PHC), which included five subcentres and Khanapur Primary Health Centre (PHC), which included eight subcentres.

### Sample size and sampling technique

The sample size was determined for the parent research project investigating the prevalence and determinants of postpartum depression among postpartum mothers. The same study population was used for the present analysis on awareness of postpartum depression.

The required sample size was calculated using the formula.

$${\mathrm{n}} = {\mathrm{Z}}^{2} \times {\mathrm{p}} \times {\mathrm{q}}/{\mathrm{e}}^{2} $$

where:

- *Z* = 1.96 (for 95% confidence interval).
- *p* = 11% (anticipated prevalence of PPD [20].
- *q* = 1 – *p* = 89%.
- *e* = 5% (margin of error).

This estimated the minimum required sample size of 150 participants. After applying a design effect of 1.5 and adjusting for a 10% non-response rate, the target sample size was increased to 250.

A total of 240 eligible postpartum mothers were approached during the study period, of whom 220 consented to participate, resulting in a participation rate of 91.7%. Due to feasibility constraints and participant availability during the study period, 220 postpartum mothers were enrolled. A balanced representation was obtained across all 13 subcentres. Although the final enrolled sample (*n* = 220) was lower than the adjusted target sample size of 250 participants, it exceeded the minimum estimated sample size of 150 participants.

A multi-stage sampling technique was used to recruit postpartum mothers. Haveli Taluka was randomly selected from the 14 Talukas in Pune District. From the 12 PHCs in Haveli Taluka, two PHCs (Khanapur and Khadkwasla) were selected randomly. All subcentres under the selected PHCs were included to ensure the geographical representation of the sample.

Participants were consecutively recruited during immunisation sessions conducted at the selected subcentres. Eligible mothers attending the sessions were approached and invited to participate in the study. For mothers who left early or were unavailable, the recruitment period was extended to additional sessions, and recruitment continued until the desired sample size was reached.

### Inclusion and exclusion criteria

Postpartum mothers between 6 weeks and 6 months post-delivery, aged 14–49 years and residing in Rural PHC catchment areas for at least 6 months were included. Mothers with prior severe psychiatric illness, those currently receiving psychiatric treatment, those with significant medical illness requiring hospitalisation or ongoing treatment, and those with stillbirth and neonatal death outcomes in the current pregnancy were excluded from the study.

### Data collection

A structured questionnaire was administered through face-to- face interview to assess the participants’ socio- demographic profiles, and awareness of PPD ( Have you heard about PPD? ), recognition of key symptoms( sadness, crying spells, loss of interest, irritability), sources of information (Accredited Social Health Activists(ASHAs), Doctors, Peers, Media) and attitudes towards help-seeking (perceiving symptoms as a “normal part of motherhood” versus an “illness requiring professional help”).

The questionnaire was developed by the authors after reviewing the relevant literature on postpartum awareness and maternal mental health. The questionnaire underwent expert review by mental health experts and public health experts to assess content validity. The questionnaire was initially prepared in English and then translated into Marathi and back-translated into English to ensure cultural appropriateness and linguistic consistency. Symptom-related terms such as sadness, irritability and loss of interest in daily activities were explained using simple locally used expressions during pilot testing and interviews to enhance the comprehension of participants with different literacy levels. A pilot test was performed among postpartum mothers from similar rural settings to evaluate the feasibility, clarity and comprehension of the administration. Necessary modifications were made based on participants’ feedback prior to the final data collection. Interviews were conducted by the primary researcher in the vernacular language (Marathi) to ensure participant understanding and consistency in data collection.

### Data analysis

Data were entered, cleaned and managed using Microsoft Excel 365 (Version 2604), before exporting to R software (version 4.1.2, 2021-11-01). Data cleaning involved checking the dataset for completeness, consistency, duplicate entries and missing values. Incomplete and missing responses were excluded from the analysis. A descriptive analysis including frequencies and percentages was performed for categorical variables. Inferential statistics were conducted using the chi-square test and Fisher’s Exact Test, wherever appropriate, to assess the association between awareness of PPD and selected socio-demographic characteristics of the participants such as mothers’ age, education level, socioeconomic status, parity, and employment status. A *p*-value < 0.05 was considered statistically significant.

Ethical approval was obtained from the Institutional Ethics Committee of Vishwaraj Hospital, Loni Kalbhor, Pune, India. (Approval No. IEC/O-64/2024). Participation was voluntary, and confidentiality was maintained. Written informed consent was obtained from all participants prior to data collection. Participants who appeared to be emotionally distressed during the interview were approached with sensitivity and were referred to the appropriate PHC healthcare provider for further evaluation and support as required.

## Results

### Socio-demographic profile of participants

A total of 220 postpartum mothers were enrolled in the study from rural areas of Haveli Taluka, District Pune, Maharashtra. Socio-demographic characteristics are presented in Table 1. The median age of participants was 26 years (IQR:23–29), about 8.2% of mothers had no formal or primary education, 55% of participants had completed secondary education, and 36.8% had higher education. Of the mothers, 89.5% were unemployed. Regarding socioeconomic status, 26 (11.81%) mothers were from Above Poverty Line (APL) households, and 194 (88.18%) were from Below Poverty Line (BPL) households. According to the National Food Security Act Guidelines in India, socioeconomic status was categorised based on the possession of government -issued Below Poverty Line (BPL) and Above Poverty Line (APL) ration cards under the Public Distribution System [21]. Nearly half of the mothers (50.5%) were primiparous.

**Table 1:** Socio-demographic and obstetric characteristics of mothers (*N* = 220)

Variables	Categories	Total *n* (%)
Mother’s age (years)	≤ 24 ≥ 25 Median: 26 (IQR: 23–29 years)	82 (37.3) 138 (62.7)
Educational status	No formal/primary Secondary Higher	18 (8.2) 121 (55.0) 81 (36.8)
Employment status	Employed Unemployed	23 (10.5) 197 (89.5)
Family type	Nuclear Joint	80 (36.4) 140 (63.6)
Socioeconomic status	BPL APL	194 (88.2) 26 (11.8)
Parity	Primiparous Multiparous	111 (50.5) 109 (49.5)
Mode of delivery	Vaginal Caesarean section	119 (54.1) 101 (45.9)
Time since delivery	6 weeks –3 months > 3 months–6 months	120 (54.5) 100(45.5)

### Awareness of PPD

Of 220 mothers, 163 (74.1%) were unaware of PPD, while only 57 (25.9%) had heard of PPD.

### Recognition of symptoms

Among mothers who were aware of PPD (*N* = 57), 28 (49.1%) were able to recognize more than three symptoms. Crying spells (38/57, 66.7%) and sadness (30/57, 52.6%) were the most frequently recognised, followed by loss of interest in daily activities (12/57, 21.1%) and irritability/ agitation (8/57, 14.1%).

### Sources of information

Social media (35/57, 61.4%) and antenatal care provider (22/57,38.6%) emerged as the main sources of information.

### Attitude towards help-seeking

Among mothers aware of PPD, nearly half (29/57, 50.9%) felt it was a normal part of motherhood, while 31.6% (18/57) considered it a health problem requiring attention; 17.5% (10/57) were unsure or did not respond. The awareness, knowledge, and attitudes regarding PPD are summarized in Table 2.

**Table 2:** Summary of Indicators related to PPD

Domain	Indicator	Frequency(*n*)	% (Denominator)
Awareness of PPD (*N* = 220)	Yes No	57 163	25.9% (57/220) 74.1% (163/220)
Recognition of more than 3 symptoms (*N* = 57)	Yes No	28 29	49.1% (28/57) 50.9% (29/57)
Sources of Information(*N* = 57)	Social media Antenatal care provider	35 22	61.4% (35/57) 38.6% (22/57)
Attitudes towards help-seeking (*N* = 57)	Normal Part of motherhood Willing to seek help Unsure/ Did not respond	29 18 10	50.9% (29/57) 31.6% (18/57) 17.5% (10/57)

### Factors associated with awareness of PPD

Inferential analysis showed a statistically significant association between maternal age and awareness of PPD (χ^2^ = 11.69, *p* < 0.001). Mothers aged ≥ 25 years demonstrated significantly higher awareness compared to younger mothers. Education status was also significantly associated with awareness of PPD (Fisher’s Exact test, *p* < 0.001), with higher awareness reported among participants with secondary and higher education levels. The study reported no statistically significant association between socioeconomic status and awareness of PPD (χ^2^ = 1.14, *p* = 0.286), employment status and awareness of PPD (χ^2^ = 1.63, *p* = 0.201), or parity and awareness of PPD (χ^2^ ≈ 0.00, *p* = 1.00). (See Table 3 for factors associated with awareness of PPD)

**Table 3:** Factors associated with awareness of PPD

Variable	Category	Aware of PPD *n* (%)	Not aware of PPD *n* (%)	Chi-square (χ^2^), *p*-value
Mother’s age (years)	≤ 24 ≥ 25	10 (12.2%) 47(34.1%)	72(87.8%) 91(65.9%)	χ^2^ = 11.69, *p* < 0.001
Educational status	No formal/Primary Secondary Higher	3 (16.7%) 20 (16.5%) 34 (42.0%)	15 (83.3%) 101(83.5%) 47 (58.0%)	*p* < 0.001*
Socioeconomic status	BPL APL	53 (27.3%) 4 (15.4%)	141 (72.7%) 22 (84.6%)	χ^2^ = 1.14, *p* = 0.286
Employment status	Employed Unemployed	9 (39.1%) 48 (24.4%)	14 (60.9%) 149 (75.6%)	χ^2^ = 1.63, *p* = 0.201
Parity	Primiparous Multiparous	29 (26.1%) 28 (25.7%)	82 (73.9%) 81 (74.3%)	χ^2^ = 0.00, *p* = 1.00

*Fisher’s exact test

## Discussion

The present study revealed that nearly three-fourths of the participants attending rural immunisation services were not aware of PPD, and less than half of the mothers who had heard of PPD were able to identify more than three symptoms of the condition. These findings indicate substantial gaps in maternal mental health knowledge and recognition of postpartum psychological symptoms within rural communities. Similar findings have been reported In India and other countries, indicating that inadequate awareness of maternal mental health is a wider public health concern. A scoping review reported a scarcity of studies assessing PPD awareness in India and highlighted the widespread misdiagnosis and under-recognition of maternal depressive symptoms [17].

Poreddi et al. [8] reported inadequate knowledge and recognition of PPD symptoms among women and families [8], indicating that limited awareness is a widespread challenge even outside rural area. Studies from Nigeria, Iran and Saudi Arabia have also reported inadequate awareness and poor recognition of PPD symptoms among women during the perinatal period [22–24]. In the present study, among mothers who were aware of PPD, crying spells and sadness were the most often recognized symptoms, while symptoms such as irritability and loss of interest in daily activities were less commonly identified. Previous studies have shown similar patterns of partial symptom recognition, with overt emotional symptoms being more likely to be noticed than the cognitive and behavioural manifestations of depression [15, 23, 25]. This pattern may reflect a limited understanding of the broader clinical presentation of PPD.

A substantial proportion of mothers in this study perceived emotional distress as a normal aspect of motherhood, which may contribute to delays in recognition and help-seeking for postpartum mental health issues. The normalisation of emotional difficulties after childbirth, sociocultural expectations surrounding motherhood, stigma around mental illness and limited family-level understanding of maternal psychological wellbeing may affect awareness and healthcare-seeking behaviour. Similar help-seeking barriers have been reported by Bilszta et al. [14], Fonseca et al. [15], Henshaw et al. [26], and Byatt et al. [27], who identified stigma, symptom normalisation, and lack of mental health literacy as barriers to timely support and management during the perinatal period [14, 15, 26, 27].

Although mothers in rural areas frequently interact with healthcare services for antenatal care, postnatal care and child immunisation, maternal mental health counselling is still poorly integrated into routine maternal and child healthcare delivery. In this study, social media emerged as a source of information among mothers who were aware of the condition.

Although ASHA workers were not formally assessed as part of the study participants, informal field interactions indicated that awareness of postpartum depression among frontline health workers may warrant further investigation. Similar knowledge gaps regarding perinatal mental health among healthcare providers and service utilisers have been highlighted in India [28]. 

Inferential analysis showed that mothers’ age was significantly associated with awareness of PPD, with older mothers showing more awareness than younger mothers. Educational status was also significantly associated with awareness with mothers with having higher education demonstrating better knowledge. Similar associations between education and mental health literacy have been reported previously [8, 29, 30]. Higher educational level may facilitate access to health information, digital media and healthcare communication.

The findings of this study underscore the need for context-specific maternal mental health awareness initiatives in primary healthcare settings. Incorporating culturally appropriate information regarding PPD into antenatal counselling, postnatal follow-up, Village Health and Nutrition Days (VHND), and immunisation sessions may enhance the early recognition of postpartum psychological distress. Strengthening ASHA- and Auxiliary Nurse Midwife (ANM) -led maternal mental health education and integrating basic awareness into existing maternal and child health programs may support timely identification, referral and help-seeking among postpartum mothers in rural communities.

### Strength and limitations

The present study contributes to the limited evidence on PPD awareness among rural postpartum women in India. The study additionally examined symptom recognition, help-seeking attitudes, and factors linked to awareness of PPD within a primary healthcare setting, delivering context-specific insights related to maternal mental health services in rural communities.

While interpreting the findings of the present study, certain limitations should be considered. As the study design was cross-sectional in nature, causal association cannot be established. Although study settings were selected using multi-stage sampling, participants were enrolled consecutively during immunisation sessions, which may have limited the representativeness of the sample and generalizability of findings beyond similar rural contexts. Awareness-related responses were self-reported, inducing social desirability bias and recall bias. The present study did not employ a validated PPD awareness scale, which may affect the standardisation of awareness assessment. The study did not include a detailed assessment of depressive symptoms using standardised tools such as the Edinburgh Postnatal Depression Scale (EPDS) or PHQ-9, which may limit our understanding of the relationship between awareness and actual symptomatology. Finally, ASHA workers were not formally evaluated as study participants; observations regarding their awareness emerged informally during field interactions and should be interpreted cautiously. As participants were enrolled only from mothers attending immunisation sessions at selected PHCs, the possibility of selection bias cannot be ruled out, and the findings may not represent postpartum mothers who do not utilise immunisation services.

## Conclusion

The present study found that awareness of PPD was low among rural postpartum mothers, with approximately three-fourths of the participants reporting that they had not heard of the condition. Symptom recognition was also poor, and social media was the most common source of information. These findings underscore the need to strengthen maternal mental health awareness within existing primary healthcare services through culturally appropriate counselling, community-based education, and enhanced ASHA/ANM-led maternal mental health communication, which may promote early recognition and timely help-seeking in rural communities.

## Data Availability

The dataset generated and analysed during the current study is available from the corresponding author on reasonable request.
